# Willingness to take responsibility: Self-sacrifice versus sacrificing others in takeover decisions during autonomous driving

**DOI:** 10.1016/j.heliyon.2024.e29616

**Published:** 2024-04-16

**Authors:** Uijong Ju, Sanghyeon Kim

**Affiliations:** Department of Information Display, Kyung Hee University, Seoul, South Korea

**Keywords:** Autonomous vehicle, Takeover, Responsibility, Video survey, Text survey

## Abstract

In Level-3 autonomous driving, drivers are required to take over in an emergency upon receiving a request from an autonomous vehicle (AV). However, before the deadline for the takeover request expires, drivers are not considered fully responsible for the accident, which may make them hesitant to assume control and take on full liability before the time runs out. Therefore, to prevent problems caused by late takeover, it is important to know which factors influence a driver's willingness to take over in an emergency. To address this issue, we recruited 250 participants each for both video-based and text-based surveys to investigate the takeover decision in a dilemmatic situation that can endanger the driver, with the AV either sacrificing a group of pedestrians or the driver if the participants do not intervene. The results showed that 88.2% of respondents chose to take over when the AV intended to sacrifice the driver, while only 59.4% wanted to take over when the pedestrians would be sacrificed. Additionally, when the AV's chosen path matched the participant's intention, 77.4% chose to take over when the car intended to sacrifice the driver compared with only 34.3% when the pedestrians would be sacrificed. Furthermore, other factors such as sex, driving experience, and driving preferences partially influenced takeover decisions; however, they had a smaller effect than the situational context. Overall, our findings show that regardless of the driving intention of an AV, informing drivers that their safety is at risk can enhance their willingness to take over control of an AV in critical situations.

## 1 Introduction

According to the definition of the level of automation by the Society of Automotive Engineers, an autonomous vehicle (AV) is the main driver in Level-3 autonomous driving; however, the driver should intervene and be responsible for driving after receiving a request from the AV in emergencies [1]. As emergencies can occur within a second during autonomous driving, it is difficult to determine whether the driver or AV is responsible for accidents that occur during takeover transition. For example, Mercedes-Benz, which has been granted permission to operate the first Level-3 AV in Germany [2], provides the driver with 10 s to take over [3], based on the United Nations Economic Commission for Europe (UNECE) regulation reports [4]. This system raises certain questions regarding accidents that occur within the 10 s given for takeover; specifically, does the driver of the Level-3 AV hold potential liability for accidents occurring promptly after a takeover request or is the driver liable only for accidents that happen after the 10-s grace period mandated by UNECE regulation reports [5]?

As Level-3 AVs are not yet fully commercialized, there are no clear laws regarding this issue and only indirect proposals exist. For instance, the United Kingdom government recommends that the person sitting in the driver's seat cannot be prosecuted for crimes directly arising from driving tasks such as violations and dangerous driving [6], implying that the liability of Level-3 AV accidents during takeover does not entirely rest on the driver and that the manufacturer could shoulder some responsibility for such accidents.

In the future, the complete commercialization without limitation of Level-3 AVs will increase debates regarding whether drivers should take over control in emergencies wherein an accident may occur before the takeover time or whether they can leave the decision entirely to the AV. This debate is even more relevant [7] as an immediate takeover may not be particularly beneficial because the driver could become over-dependent on the AV and disconnected from driving monitoring [8]; therefore, the takeover process can cause problems such as a loss of situational awareness [9] and degradation of driving performance, and the takeover could transfer the responsibility for the accident from the AV to the driver. According to previous research, 94% of drivers want to be alerted when an emergency arises, but only 64% want to be asked to approve the AV's decision or take over control of the car [10]. This suggests that while drivers generally want to be aware of emergencies, preferences for or against manual driving in critical situations vary. This result can be explained by the public choice theory, which states that a shifting of responsibility can indeed be a critical element in making delegation decisions [11] and decision-makers may find it easier to alleviate themselves of guilt when they delegate tasks to machines, as opposed to delegating them to other individuals [12]. This is supported by previous research that some drivers prefer to leave decisions to their AV because they do not want to take responsibility or fear feeling guilty about the future consequences of their decisions. For example, a previous simulation study reported that participants prefer to adopt a suggestion from an AV rather than drive manually to avoid the intensity and stress of driving in a critical situation [13]. An online survey also showed that people prefer to delegate decisions to artificial intelligence (AI) over humans when those decisions entail losses [14]. These findings show that some drivers may indeed prefer to leave decisions to AVs to avoid responsibility. Other studies, however, suggest that drivers may prefer to be in control due to safety concerns or because they do not want to leave a potential life-or-death decision to a machine. Safety concerns are one of the main worries of respondents to surveys on autonomous driving [15]; some think autonomous driving is not as safe as manual driving [16] and prefer to have the option of taking over and driving manually, even when relying on a fully autonomous car [17,18]. Even in situations where a semi-AV would make the right choice, some drivers prefer to take over [10]. This can be supported by the principle of self-preservation, which is defined as optimizing one's survival likelihood within a finite time frame [19]. According to this principle, manual driving can provide maximum utility for survival by increasing predictability and control. Additionally, previous studies have found that people are averse to machines making moral judgments in an emergency [20] and to algorithms in general, even though machines do outperform humans [21]. Thus, while some studies imply that drivers may prefer manual control regardless of the intention of AVs, inconsistencies in previous research, alongside a conflict between the public choice theory and self-preservation principle, suggest that in level-3 automated driving, some drivers may delay takeover to avoid the guilt and responsibility associated with potential accidents. However, several recent AV accidents have shown that when facing unexpected situations, AVs may not operate safely [22,23], and intentionally taking over late could increase the severity of accidents—even if they are impossible to avoid. To solve this problem, in the present study, we aim to investigate the factors influencing takeover decisions in critical situations to explore ways to prevent intentionally late takeovers. To achieve this goal, we investigate several potential factors that influence takeover decisions, including the situational context (e.g., climate and road conditions enhance the intention to take over rather than the emotional states of drivers) [24] and the situational assessment of a critical situation, which varies across scenarios and influences the preferences for autonomous or manual driving [10]. Situational factors also influence the driver's behavior related to takeover, and information about an AV's intended actions in critical situations enhances the driver's situational awareness and takeover quality [25,26].

While situational factors have a great impact on takeover decisions [10], driver characteristics, including sex, age, and experience, could also play a role. Studies on the effect of the driver's sex on takeover decisions show that only negative emotional states influence takeover intention across sexes [24]. However, other research on takeover behaviors has produced inconsistent results: male participants have been found to cause fewer crashes than female participants during emergency takeovers [27], but female participants have also shown slightly better takeover stability [28]. The findings on age are similarly inconsistent, with those aged under 20 showing a lower willingness to take over than older age groups [24]. Another study suggested that age does not influence the takeover time or decision-making after the takeover, and takeover quality is not significantly different between young and old-aged drivers [29], while younger adults respond faster to takeover requests and make quicker decisions than older drivers [30]. By contrast, driving experience has been found to clearly influence takeover stability and adaptability [31], with experienced drivers performing better [32,33], while bad driving styles aggravate driving behavior after takeover [34].

Previous studies have also demonstrated that the preferred driving style of the AV influences the takeover decision in critical situations; for example, if an AV's driving style contradicts the driver's preferences, the driver tends to take over more frequently [35]. Thus, the preferred driving style of the AV can be used as a factor to predict the driver's takeover decision [36] and the takeover decision of a driver is useful for predicting their driving style preferences [37].

While many prior studies have already reported several factors associated with takeover decisions [[24], [25], [26], [27], [28], [29], [30], [31], [32]], to the best of our knowledge, no studies have investigated how a driver's willingness to take over changes in emergencies when the target at risk changes, which is crucial for preventing potential accidents caused by no takeover or decision conflict between the AV and driver [24]. To address this issue, in the present study, we designed a dilemma in which it was not possible to avoid accidents based on the well-known autonomous driving dilemma, the “moral machine experiment” [38,39], which requires participants to choose between two alternatives in an emergency. We modified this dilemma to allow drivers to choose between self-sacrifice and sacrificing others; in other words, the takeover decision could only change the sacrificed target from the driver to pedestrians or vice versa. Additionally, we informed drivers of the driving intention of the AV to ensure that the takeover decision did not guarantee the prevention of an accident to make drivers consider not taking over; moreover, to make participants clearly recognize emergencies, we used video and text surveys to investigate takeover decisions during autonomous driving. Given the fact that the AV can also choose to sacrifice pedestrians to save the driver, we additionally investigated whether drivers would show a willingness to take over even if they knew that leaving the decision to the AV would sacrifice pedestrians instead of themselves. A previous study showed that those who chose autonomous driving felt less responsibility than those who chose manual driving [40]; thus, we first hypothesized that when an AV shows the intention to sacrifice pedestrians, the proportion of those who choose autonomous driving increases. Additionally, as previous studies have shown that the proportion of people choosing self-sacrifice decreases in autonomous driving [41] and that people prefer to buy cars that prioritize drivers [38], our second hypothesis was that participants’ takeover decisions increase when they know that they will be sacrificed if they leave the decision to the AV.

In addition, as mentioned above, because the driver's personal characteristics could influence the takeover decision, we investigated the participants’ characteristics to find potential relationships between driver characteristics and the takeover decision. Finally, as mentioned above, individuals’ preferred driving style could reflect their general attitudes toward AV; thus, we hypothesized that preferred driving style could be associated with takeover intention. In summary, we established the following four hypotheses for the present study: First, when takeover intention matches the AV's driving intention, the proportion of those who leave the decision to the AV increases when the AV shows an intention to sacrifice pedestrians. Second, takeover intention during autonomous driving increases when an AV's driving intention results in self-sacrifice. Third, the driver's personal characteristics significantly influence the takeover decision, although their influence is lower than that of the AV's driving intention. Fourth, there is a relationship between the takeover decision and preferred driving style of the AV.

## 2 Materials and methods

### 2.1 Sample size and participants

All participants were university students recruited via five different university (Kyung Hee university, Korea university, University of Seoul, Hankuk university of foreign studies, Yonsei University) community sites located in the same city, which meant that offline experiments could potentially be conducted in the future. To prevent population poisoning, we asked participants to apply for the experiment using their university e-mail addresses to prevent them from taking part several times. Successfully recruited participants were sent a Google Docs link and had 3 days to complete the survey. Those who did not finish the survey within 3 days were excluded, and we recruited additional participants following the same procedure to recruit the target numbers.

The required sample size for our survey was determined with a G* power analysis [42] based on chi-square tests. We referenced a previous manual driving study [43], which asked participants to choose between self-sacrifice to save pedestrians and self-sacrifice to save trees, similar to the present study wherein the AV's choice of direction could result in self-sacrifice or sacrificing others. Chi-square tests yielded an effect size of 0.23 for our previous study; thus, we set an effect size of 0.23, an error probability of 0.025 (0.05/2 for two experiments), and a power of 80%, with 1 degree of freedom (auto or manual), and determined that we needed at least 180 participants for each experiment.

We thus recruited 250 participants for the video survey (106 males, 18–34 years of age, mean age 23.1 years [SD = 3.3]) and 250 participants for the text survey (106 males, 18–37 years of age, mean age 23.0 years [SD = 3.1]), and participants were independently recruited at different time points. All participants provided informed consent during the experiment.

### 2.2 Stimulus

To provide the same emergency to all participants in the video survey, we used virtual reality technology to create an autonomous driving simulation and record the entire course. The simulation was based on Unity3D 2019.1.11 (a 3D game development engine developed by Unity Technologies in San Francisco, USA), with the base driving environment taken from the Unity Asset Store (https://www.assetstore.unity3d.com/en/#!/content/10) and the waypoint AI system from Unity Learn (https://learn.unity.com/tutorial/waypoints-1?uv=2019.1). A total of 74 waypoints were inserted into the course such that one could be reached from the next within 2 s, allowing for smooth driving. Additionally, based on a previous study that had participants drive the same course [43], we restricted the maximum driving speed to 100 km/h to simulate normal manual driving conditions and provide participants with sufficient time to recognize emergencies. It took our AV 2 min and 20 s to finish the course, and 1 min and 45 s to reach the location where the emergency occurred. Additionally, we purchased the Unity AVPro Movie Capture plugin (https://assetstore.unity.com/packages/tools/video/avpro-movie-capture-basic-edition-221916) and recorded videos at a resolution of 1680 × 1052 pixels and a frame rate of 30.

### 2.3 Experimental design (video survey)

The present study used a two-group (video and text) quasi-experimental design to compare decision-making between text and video surveys. To ensure that participants had sufficient autonomous driving experience before they were exposed to a critical situation, we conducted three training sessions and one test session that consisted of different tasks (see below).

The video survey driving course contained four forks where the AV had to choose the right path to not drive the car off the cliff. Auditory warnings providing directions were presented to participants (e.g., a voice saying “cliff to the right” in Korean), followed by the same voice stating which direction the car would take (e.g., “car will follow the road to the right”) to make participants aware of the decision the AV had taken.

In the first training video, there were no obstacles on the course and participants heard two training commands instructing them to change the driving mode (i.e., “change driving mode to manual” and “change driving mode to auto”); they also heard one cliff warning and were informed about the path that the AV had decided to take before they entered the fork. After the first video ended, participants were asked how many times the voice had instructed them to change the driving mode from auto to manual to assess their situational awareness. In the second training video, multiple obstacles were placed on the course to demonstrate the AV's ability to avoid obstacles; otherwise, the course and task were identical to what participants had experienced in the first training video. After watching the second video, participants were asked how many obstacles had appeared on the road. In the third and last training session, participants followed the same course as in the second experiment until they reached the last fork, where the road they had to take to stay on the cliff (i.e., the “safe” road) was blocked with rocks, leaving only a small gap for the car to pass through. The voice informed the participant about the path the car had chosen (e.g., “car will follow the left/right road”), and the video then showed the car avoiding the rocks and falling off the cliff. The aim of the third training session was to demonstrate to participants that the car would always follow the road they had been informed it would take and that it may take the decision to fall off the cliff. After watching the third training session, participants were asked to compare the second and third training sessions and report any differences they had observed (see Table 1 for the characteristics of training and test videos and all the questions asked during the training sessions).

**Table 1:** Questionnaire to investigate situational awareness during the training phase and characteristics of the training and test videos.

Phase	Video length	Questionnaire for assessing situational awareness	Specific characteristics of the course and the event
Training 1	2 min 20 s	How many times did a voice inform you to change to manual driving?	N/A
Training 2	2 min 20 s	How many obstacles were on the road?	Obstacle on the course
Training 3	2 min	How did the second and third training sessions differ? (free-text answer)	Safety road blocked with rocks
Test	1 min 50 s	N/A	Safety road blocked with pedestrians and vehicles

**Fig. 1:**
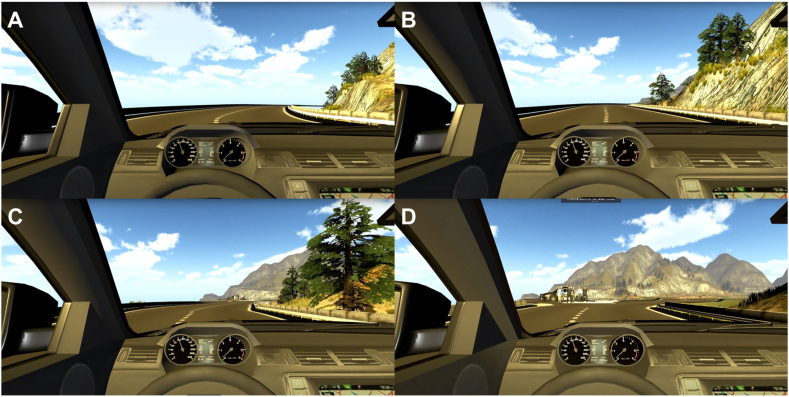
Screenshot of the emergency participants were confronted with during the test session. (A) 12 s before the event (can turn a corner), (B) 8 s before the event (can slightly observe the pedestrians), (C) 4 s before the event (can clearly observe the pedestrians and vehicle), and (D) final situation with the video stopped (can clearly observe the pedestrians, fork, and vehicle).

After they had completed all three training sessions, participants were informed that the next video was a test that would be similar to the training tasks. In the test video (as in the last training session), participants arrived at the final fork and the voice informed them that the car would follow the road that led down the cliff (“car will follow the left/right road”). Then, a group of pedestrians appeared and blocked the safe road, and participants were once again informed that the car was planning to take the road that led down the cliff (see Fig. 1(A)–(D) for the procedure in the event situation). Participants understood that the car would fall off the cliff if the driver chose to rely on autonomous driving, but also that the driver could override the AV's decision, drive manually, and drive into the pedestrians to follow the other road. The test video finished before the car went either way, however, and participants were instead asked what they would decide in such an emergency (Table 2, Q1-A; video footage of the event can be found in the “AV Study” folder on the Autonomous driving project's OSF site: https://osf.io/qdafp).

**Table 2:** Questionnaire to investigate emergency decision-making in the video and text survey.

Q1	(A) The right road at the fork leads down a cliff; hitting the brakes and stopping the car before it falls off the cliff is impossible at the current driving speed (100 km/h). If you switch to the manual driving mode and choose the road to the left instead, you will survive but the pedestrians will be sacrificed. If you follow the right road, you can save the pedestrians but you will die instead. What would you do if you were the driver? Answer: 0: Rely on autonomous driving; 1: Takeover and follow the left road; 2: Takeover and follow the right road. (B) You are traveling in a conditionally automated car (autonomous driving but the driver can take over when needed) in a rural area; there are no other vehicles around. While you are relying on autonomous driving, you hear a warning that at the next fork, the right road will lead down a cliff. After this, you see people (more than five) blocking the left road at the fork and your AV informs you that the car will follow the road on the right. The road at the right of the fork leads down the cliff; hitting the brakes and stopping the car before it falls off the cliff is impossible at the current driving speed (100 km/h). If you switch to manual driving mode and choose the road to the left instead, you will survive but the pedestrians will be sacrificed. If you follow the right road, you can save the pedestrians but you will die instead. What would you do if you were the driver? Answer: 0: Rely on autonomous driving; 1: Takeover and follow the left road; 2: Takeover and follow the right road.
Q2	**This time, your AV informs you that it will follow the left road (which means you will be saved but the pedestrians will be sacrificed). What decision would you make if you were the driver? Answer 0: Rely on autonomous driving; 1: Takeover and follow the left road; 2: Takeover and follow the right road.**

Questions in bold font were presented in the video survey as well. 0, 1, and 2 represent the possible responses the participants could choose.

### 2.4 Experimental design (text survey)

In the text-based survey, we used text descriptions to explain the same environment and events to our participants (e.g., they are in a conditionally automated car, driving on rural roads; the driver has to intervene when needed). They then answered the same questions assessing their decision-making behavior during the described emergency (see Table 2, Q1-B), and the same questions assessing their driving style preferences and attitudes toward autonomous driving in general (Table 2, Q2, Table 3). Across both surveys, there were no specific time limits, although participants were required to complete the questionnaire without any delay.

**Table 3:** Questionnaire related to autonomous driving in general.

Q1	If you buy an AV and choose to rely on it in emergencies, what setting would you prefer? 1: Always prioritize the driver's safety 2: Sacrifice the driver if many pedestrians can be saved.
Q2	If you buy an AV, what setting would you prefer for emergencies? 1: Give control to the driver 2: Give control to the AV.
Q3	Who has the responsibility for an accident if it occurs due to a judgment conflict between the driver and the AV? 1: The driver 2: The AV.
Q4	Do you intent to buy an AV in the future? 1: Yes 2: No.

### 2.5 Questionnaire related to the event

In both surveys, after asking for participants’ decision-making in the emergency, a question was posed, asking them to assume a situation wherein the autonomous car had chosen to drive into the pedestrians. Additionally, more questions assessed the participants’ driving style preferences and attitudes toward autonomous driving to identify the associations between these factors and decision-making during the emergency (see Table 3). Based on decision-making and the questionnaire related to the event, we applied a chi-square test to assess decision-making differences between the different survey platforms and event situation.

### 2.6 Experimental procedure

To participate in the video survey, participants were first asked to ensure that sound was enabled on their desktop or laptop computer. They then followed the Google Docs link to the survey, read the instructions, and provided some personal information related to the experiment. Subsequently, they watched several YouTube videos through a link embedded in the survey. After each of the three training videos, participants were asked a question to make sure that they were watching the video with the sound enabled and to evaluate their situational awareness. Participants were informed that those who answered all three questions incorrectly would be excluded from the analysis; in our study, none of the participants failed the evaluation questions. During the test video (following the training), the video stopped immediately before the car went either way at the last fork. Participants were then asked questions related to the emergency (see Table 2, Q1-A, Table 2, Q2) and the experiment (Table 3).

**Fig. 2:**
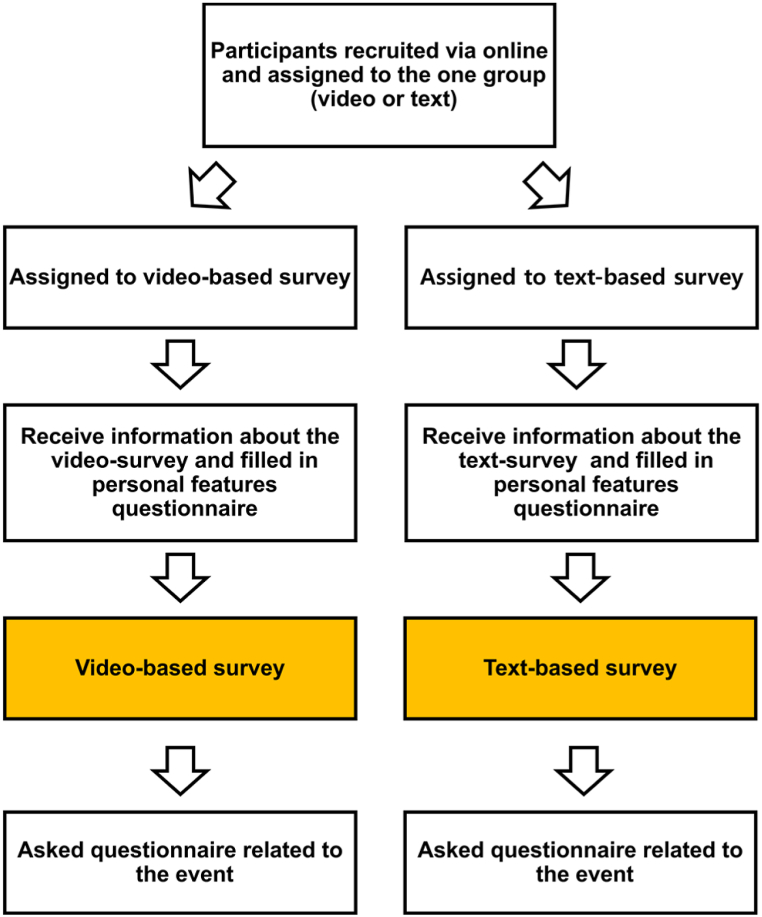
Overall experimental procedure for the video- and text-based surveys.

The text survey followed the same procedures (application via university e-mail, Google Docs sent via the same address). The only difference between the video and text surveys was that no videos were presented in the latter; otherwise, participants went through the same explanations and instructions to ensure both surveys were as similar as possible. The emergency that occurred during autonomous driving was described in the text (Table 2, Q1-B) and followed by the questionnaire questions (Table 2, Q2, and Table 3), and the overall experimental procedure of the present study is shown in Fig. 2.

## 3 Results

### 3.1 Participant characteristics between survey samples

Participant characteristics were analyzed using chi-square tests and independent sample t-tests to assess potential differences between the two survey groups (see Table 4). We found no significant differences in sex (chi-square test, χ2 = 0, p = 1.0), driving license possession (chi-square test, χ2 = 0.35, p = 0.555), annual driving distance (Wilcoxon rank-sum test, p = 0.534), or age (independent t-tests, t(498) = 0.40, p = 0.693) between the two participant samples tested on different survey types (Additional analyses to investigate the differences between survey methods for the takeover decision are included in the supplementary information, which can be found in the folder “AV study” on the Autonomous driving project's OSF site: https://osf.io/qdafp/).

**Table 4:** Driver characteristics according to survey type.

	Text survey (SD)	Video survey (SD)
Sex – Male/Female	106/144	106/144
Driving license – Yes/No	180/70	174/76
Driving distance – km (SD)	1813.22 (9445.60)	817.34 (2894.96)
Age (SD)	23.0 (3.29)	23.1 (3.05)

### 3.2 Takeover decision changes regarding who will be sacrificed

**Fig. 3:**
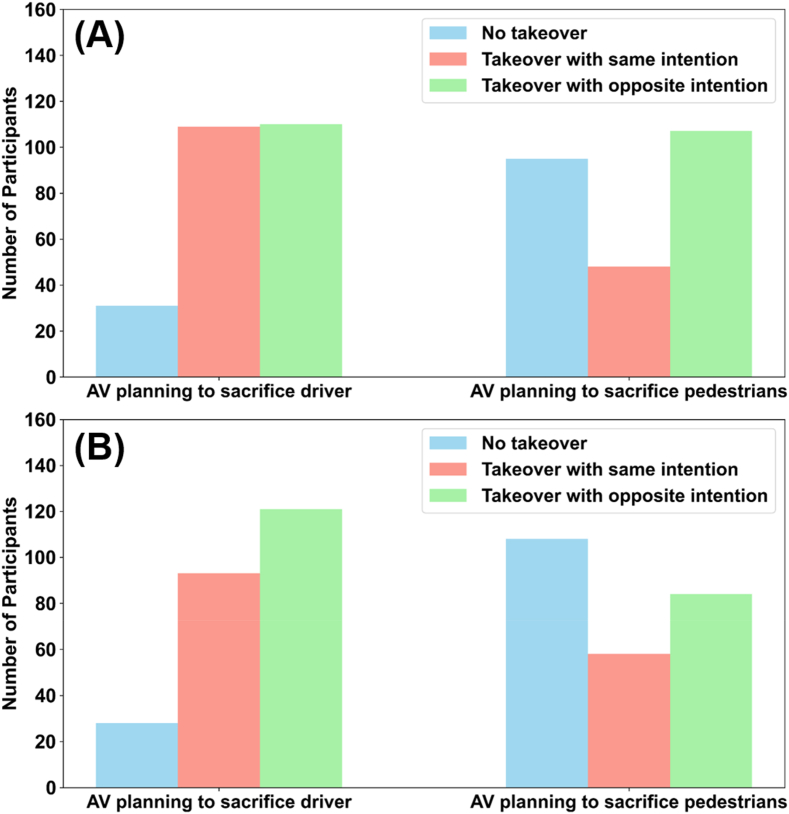
Takeover decisions according to survey type and situation (A) Video survey (B) Text survey.

The main goal of the present study was to investigate how the takeover decision changes when the AV-chosen path would result in sacrificing either the driver or the pedestrians. Based on the chi-square test, our results showed that participants chose to take over significantly more when the AV's chosen path would result in sacrificing the driver, in both the video (chi-square test, χ2 = 43.460, p < 0.001) and the text survey (chi-square test, χ2 = 64.641, p < 0.001). Additionally, when participants’ intentions were in line with the AV's chosen path, they relied on autonomous driving significantly more often when the AV's chosen path would result in sacrificing the pedestrians than when it would result in sacrificing the driver, in both the video (chi-square test, χ2 = 56.183, p < 0.001) and the text survey (chi-square test, χ2 = 49.328, p < 0.001). In summary, participants preferred to take control when the AV's decision would result in sacrificing the driver, but preferred to rely on autonomous driving when the AV chose to sacrifice the pedestrians (see Fig. 3 and Table S1).

### 3.3 Relationship between takeover decisions and driver characteristics

**Fig. 4:**
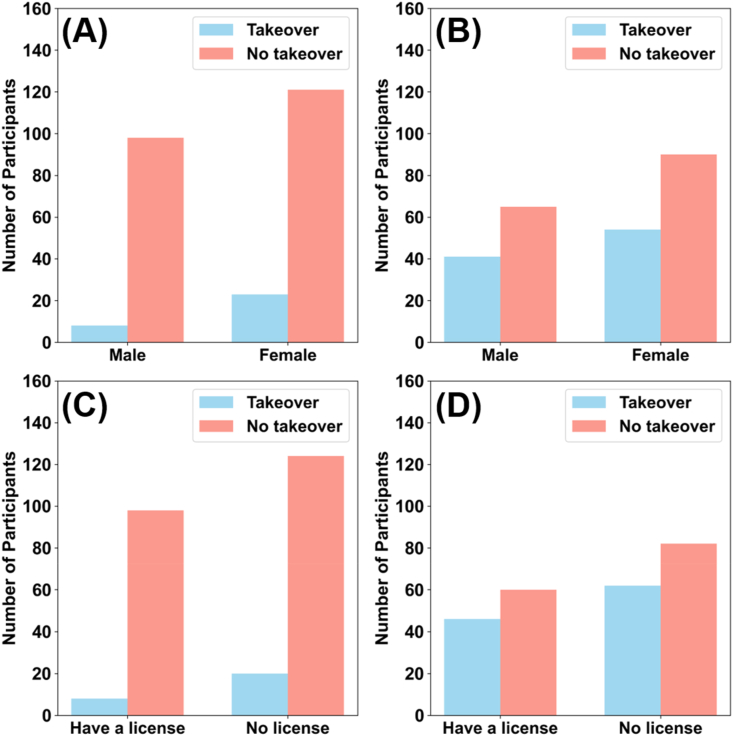
Role of sex and driving license possession in takeover decisions. (A) AV planning to sacrifice driver – video survey (B) AV planning to sacrifice pedestrians – video survey (C) AV planning to sacrifice driver – text survey (D) AV planning to sacrifice pedestrians – text survey.

**Fig. 5:**
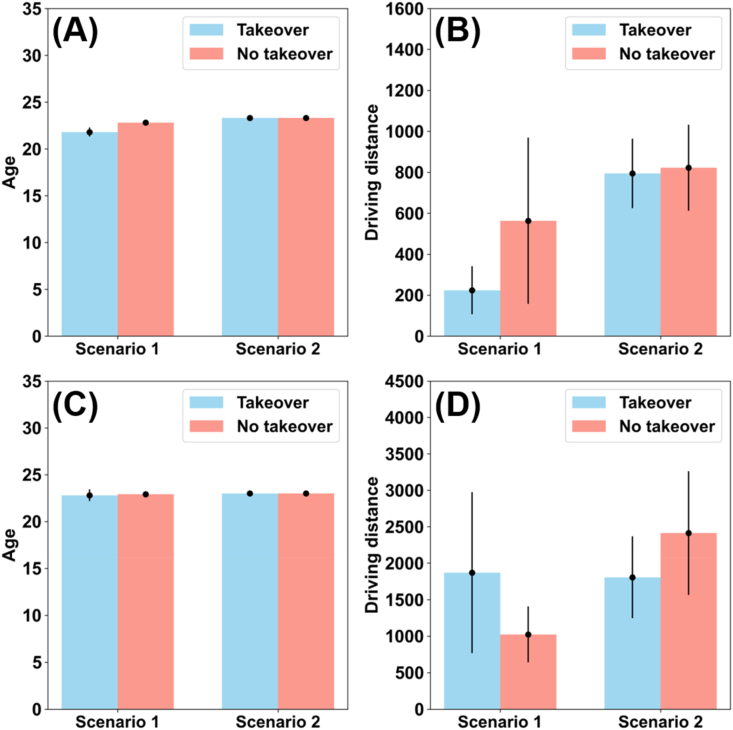
Role of age and driving experience in takeover decisions. (A) Video survey with age (B) Video survey with driving distance (C) Text survey with age (D) Text survey with driving distance. Scenario 1: AV planning to sacrifice driver, Scenario 2: AV planning to sacrifice pedestrians. Errorbar represents standard error of the mean.

Next, we conducted chi-square tests and independent sample t-tests to assess the influence of driver characteristics on takeover decisions. When the AV's chosen path would result in sacrificing the driver in the video survey, takeover decisions significantly differed between sex (chi-square test, video: χ2 = 3.990, p = 0.046; text: χ2 = 2.469, p = 0.116) and age (independent sample t-tests: video: t(248) = 2.508, p = 0.013; text: t(248) = 0.311, p = 0.756) in that female and younger participants chose to rely on autonomous driving more often (see Fig. 4-A, Fig. 5-A, and Table S2, S3-A). By contrast, there were no significant differences in driving license possession (chi-square test, video: χ2 = 3.990, p = 0.046; text: χ2 = 0.268, p = 0.604) and annual driving distance (independent sample t-tests: video: t(248) = 1.257, p = 0.210; text: t(248) = 0.039, p = 0.969) between participants in the two surveys (see Fig. 4-B, Fig. 5-B and Table S2, S3-B). When the AV's chosen path would result in sacrificing the pedestrians, we observed no significant influence of any participant characteristics on takeover decisions in either survey, neither for sex (chi-square test, video: χ2 = 0.036, p = 0.849; text: χ2 = 0.003, p = 0.957) nor for driving license possession (chi-square test, video: χ2 = 3.005, p = 0.083; text: χ2 = 3.695, p = 0.055), age (independent sample t-tests: video: t(248) = 1.136, p = 0.257; text: t(248) = 0.447, p = 0.655), or annual driving distance (independent sample t-tests: video: t(248) = 0.839, p = 0.402; text: t(248) = 1.347, p = 0.179) (see Fig. 4, Fig. 5D, and Table S2-B, S3-B). In summary, only sex and age significantly influenced the takeover decision; however, these influences were smaller than those of informing about the sacrificed target.

### 3.4 Relationship between takeover decisions and the preferred driving style of the AV

Finally, we used chi-square tests to investigate the relationship between takeover decisions and the preferred driving style of the AV. When the AV's chosen path would result in sacrificing the driver, participants who chose to rely on autonomous driving agreed significantly more often that AVs should prioritize saving the driver in the text (but not the video) survey (video: χ2 = 3.468, p = 0.063; text: χ2 = 8.651, p = 0.003). We observed no significant influence of any other AV-related preference, neither for driving priority in an emergency (video: χ2 = 3.666, p = 0.056; text: χ2 = 0.398, p = 0.528), nor for responsibility for accidents (video: χ2 = 0.009, p = 0.925; text: χ2 = 2.170, p = 0.141) or willingness to buy AV cars (video: χ2 = 0.007, p = 0.934; text χ2 = 0.417, p = 0.518) (see Table 5–A).

**Table 5:** Role of the preferred driving style of the AV in takeover decisions.

		(A) AV intends to sacrifice the driver			(B) AV intends to sacrifice the pedestrians		
	Decision		No takeover	Takeover		No takeover	Takeover
Prioritize driver vs. pedestrians	Driver/pedestrians (video)		16/15	150/69		86/9	80/75
	Driver/pedestrians (text)		14/14	169/53		96/12	87/55
	Total		30/29	319/122		182/21	167/130
Give control to driver vs. AV	Driver/AI (video)		26/5	205/14		83/12	148/7
	Driver/AI (text)		23/4	199/24		90/18	133/9
	Total		49/9	404/38		173/30	281/16
Give responsibility to driver vs. AV	Driver/AI (video)		16/15	115/104		50/45	81/74
	Driver/AI (text)		19/9	118/104		64/44	73/69
	Total		35/24	233/208		114/89	154/143
Buy AV car in the future	Yes/no (video)		23/8	164/55		75/20	112/43
	Yes/no (text)		25/3	188/34		97/11	116/26
	Total		48/11	352/89	172/31		228/69

Second, when the AV had chosen to drive into and sacrifice the pedestrians, participants who chose to rely on autonomous driving agreed significantly more often that AVs should prioritize saving the driver in both surveys (video: χ2 = 39.977, p < 0.001, text: χ2 = 23.857, p < 0.001) and that driving priority should be given to the AV in an emergency (video: χ2 = 5.524, p = 0.019; text: χ2 = 6.793, p = 0.009). There were no significant differences in participants’ views on accident responsibility (video: χ2 = 0.003, p = 0.954; text: χ2 = 1.526, p = 0.217) or willingness to buy AV cars (video: χ2 = 1.398, p = 0.237; text: χ2 = 3.211, p = 0.073) (see Table 5–B).

In summary, participants’ decisions were more in line with their preferred driving style of the AV during an emergency in situations wherein the AV had chosen the path that would result in sacrificing the pedestrians and saving the driver.

## 4 Discussion

The present study used video and text surveys to investigate takeover decisions in conditional autonomous driving when participants were informed which path their AV would take. We found that in both surveys, participants chose to drive manually significantly more often when the AV's chosen path would result in sacrificing the driver, while reliance on autonomous driving significantly increased when the AV decided to sacrifice the pedestrians.

### 4.1 Dependence of takeover decisions on the sacrificed target

The first and second hypotheses of our study were that takeover decisions differ depending on whether they result in self-sacrifice or sacrificing others and that when takeover intention matches that of the AV, the proportion of those who choose autonomous driving increases when the AV shows an intention to sacrifice pedestrians. We found that 88.2% of participants chose to take over when the AV's chosen path would result in sacrificing the driver and saving the pedestrians; additionally, even if participants’ intentions were in line with those of the AV, we found that 77.4% of drivers chose to take over when the AV had decided to sacrifice the driver and save the pedestrians. These results imply that when a driver is aware that their safety is threatened, the likelihood of takeover increases regardless of the driving intention of the AV, which could be used to enhance drivers’ takeover willingness and prevent late or delayed takeover. This finding was supported by a previous study, which found that drivers may want to take over even when an AV would make the “right” choice [10] and that people are generally averse to machines making moral judgments [20].

Contrary to previous results, when the AV's chosen path would result in saving the driver and sacrificing the pedestrians, our participants chose to drive manually significantly less often, with only 59.4% choosing to take over; moreover, even if participants’ intentions were in line with that of the AV, only 34.3% of drivers chose to take over when the car had decided to sacrifice the pedestrians and save the driver. These results show that many drivers want to leave the decision to the AV and avoid responsibility for accidents if self-safety is guaranteed, which is in line with a previous study in which participants were reluctant to make decisions with potentially deadly consequences for others, except when omissions would lead to self-sacrifice [44]. Furthermore, survey participants have also been found to give less responsibility to drivers relying on an AV than those choosing to drive manually in critical situations [40]. In short, informing drivers to guarantee their safety could delay the takeover decision, which could reduce the severity of accidents caused by drivers.

Overall, the results support the first and second hypotheses that participants prefer manual driving and have a willingness to take responsibility when the informed path comprises sacrificing themselves and that the proportion of those who choose autonomous driving increases when the informed path does not harm the driver. These results imply that self-preservation makes people choose takeover regardless of the driving intention of the AV, and if the self-preservation instinct is satisfied, many drivers choose to delegate responsibility to the AV.

### 4.2 Influences of driver characteristics on takeover decisions

Our third hypothesis was that driver characteristics significantly influence takeover decisions. We found this to be true only in the video survey; when the chosen path resulted in sacrificing the driver, sex and age significantly influenced participants’ decisions, with female and younger participants relying more often on autonomous driving. A potential explanation for this finding is the higher emotional arousal evoked by the self-sacrifice situation during the video survey, which could have affected men and women as well as younger and older participants in different ways. Previous studies have shown that visually presented or desktop virtual reality dilemmas induce higher emotional arousal than text surveys [45,46] and that requiring a participant to commit harmful actions against others enhances their emotional arousal in moral conflict situations compared with when they can rely on autonomous driving [44,47]. Accordingly, as female participants show higher emotional arousal when watching videos that induce specific emotions [48] and significantly higher self-focused emotional reactions in general [49,50], we assume that our female participants avoided manual driving in critical situations to evade the higher intensity and stress compared with relying on the AV [44]. Similarly, the relatively low emotional regulation abilities of young adults [51] may influence their takeover decisions, although emotional arousal differences are inconsistent across age groups [52]. Another possible explanation for our findings is the generally higher acceptability of AVs among young adults [53,54], who are excited to use new technology associated with sensation-seeking [55]; this might make them more likely to leave a decision to the AV when their intention matches that of the car. Overall, these results partially support the third hypothesis that driver characteristics influence takeover decisions when a situation is emotionally arousing; however, the influence of driver characteristics was smaller than that of the situational context that endangered the drivers. These results suggest that if future mobility systems can recognize driver characteristics and assess physiological signals in real time, they also could help in predicting takeover decisions.

### 4.3 Influence of the preferred driving style of the AV on takeover decisions

Our fourth hypothesis was that there is a relationship between takeover decisions and the preferred driving style of the AV. We found that when the AV's chosen path resulted in sacrificing the pedestrians, participants who chose to rely on autonomous driving agreed significantly more often, in both surveys, that real-world AVs should prioritize drivers and that control should be given to the AV in emergencies. By contrast, when the AV had chosen to sacrifice the driver, only participants in the text survey agreed to give control to real-world AVs. These results showed that takeover decisions were more closely associated with participants’ preferences for real-world autonomous driving when the AV intended to save the driver. Differences in the perceived significance of the sacrifice to be made might explain this finding. A previous study found that when omissions resulted in self-sacrifice and participants could choose between two alternative options to sacrifice either one or three pedestrians, nobody opted for omission over action [44]. However, another study showed that when self-sacrifice could save five or more pedestrians, participants demonstrated a consistent willingness to sacrifice themselves [56]. These results suggest that drivers consider giving their own lives to save others, which is a higher form of sacrifice than risking the lives of others for their own; therefore, general preferences toward autonomous driving do not reflect takeover decisions well when omission leads to self-sacrifice. By contrast, views on who is responsible for AV accidents stemming from decision conflicts and participants’ willingness to buy AVs in the future were not significantly associated with takeover decisions in our sample. This might stem from the fact that it was not possible to avoid accidents altogether in our experiment and that takeover decisions were therefore only made to choose the more valuable option. Additionally, the critical situation was not the AV's fault and takeover decisions were not associated with general views on accident responsibility. Overall, these results partially support our fourth hypothesis that the preferred driving style of the AV and priorities regarding whom to save in an emergency are, at least to some extent, associated with takeover decisions in critical situations.

These results imply that individual preferences or driving styles could be considered when setting the driving style of a level-3 AV, to minimize potential conflicts between the AV and drivers that could occur during an emergency.

### 4.4 Study limitations

This study has some limitations that warrant future work. First, our survey did not assess actual takeover behavior; therefore, there will be a gap between the results of this study and actual decisions in real-world driving. However, owing to safety issues, it is almost impossible to test critical takeover scenarios in the real world and we believe that our study can be used as a baseline to investigate moral conflicts. In future work, to enhance the ecological validity of takeover decisions in moral conflict situations, we will use a virtual reality setup to present participants with an interactive environment. Second, our study sample was limited in age, as we recruited only young adults, which limits the generalizability of our findings. Previous studies have shown inconsistent results, with one reporting that older adults show a tendency to not harm others rather than choose the best for the majority [57], while another study found that older adults show higher acceptability of egoistic algorithms than middle-aged groups [58]. Future studies should use surveys similar to the ones employed here to further clarify the influence of age. Third, our participants answered the questions in a fixed sequence, which may have induced an order effect. However, previous studies have shown order effects in online surveys when questions were asked on separate pages [59]. In our survey, questions were presented on the same page one after the other, and we therefore assumed that the potential influence of order was small. However, future studies should take such potential confounders into account. Finally, because we used an online survey design, emotional arousal and other physiological responses could not be recorded. Future studies should conduct experiments offline and simultaneously record physiological measures (e.g., heart rate, skin conductance) to better investigate emotional arousal differences between participants in video and text surveys.

## 5 Conclusions

We conducted a video- and text-based survey experiment to investigate takeover decisions when an AV's chosen path results in either self-sacrifice or the sacrifice of pedestrians. Our findings showed that takeover decisions significantly differed depending on who the AV chose to sacrifice and that driver characteristics as well as the preferred driving style of the AV partially influenced participants’ decision-making. Our study contributes empirical evidence on how to get drivers to voluntarily take over in Level-3 autonomous driving; this will become especially important when autonomous driving becomes as safe as human driving [7] and Level-3 AVs become commercially available without limitations on the driving context. Considering a previous study showing that the driving context affects preferences for AV decisions [10] and the present findings demonstrating that the driving context affects the driver's intention to take over, cars should come with personalizable intelligent systems and takeover decisions should be fostered by informing drivers that the emergency affects the safety of drivers. For instance, in future AVs, the warning sound for a takeover request could use a looming sound to enhance the perceived risk of drivers who showed effectiveness in emergency braking [60]; additionally, information about the AV's driving intention, when only the AV's decision influences the safety of the driver, could be provided to enhance their situational awareness and willingness to take over without notifying the driver of the AV's driving intention when safety is guaranteed to prevent drivers from leaving the decision to the AV. Additionally, the findings of the present study suggest that in the future, even in Level-4 autonomous driving, AVs should be designed to hand over control to the driver when decisions are directly related to their safety; if the driver does leave a decision to the AV, they should be asked to allow the car to decide on its own even in life-threatening situations. Overall, we expect our work to inspire the development of intelligent takeover systems that ensure intuitive and predictable autonomous driving through clear communication between the driver and car.

## Ethics statement

All study procedures were approved by the local ethics committee of Kyung Hee University (KHSIRB-21-322(EA)).

## Data availability statement

The data related to this study have not been stored in a publicly available repository; however, they will be made available upon reasonable request.

## Funding

This study was supported by three grants from the 10.13039/501100003725National Research Foundation of Korea (NRF-2020R1I1A1A01057915, 2022R1F1A1060046, and RS-2023-00242928).

## CRediT authorship contribution statement

**Uijong Ju:** Writing – original draft, Visualization, Project administration, Methodology, Formal analysis, Data curation, Conceptualization. **SangHyun Kim:** Writing – review & editing, Methodology, Investigation.

## Declaration of competing interest

The authors declare that they have no known competing financial interests or personal relationships that could have appeared to influence the work reported in this paper.
