# Supplementary material for: Willingness to take responsibility: Self-sacrifice versus sacrificing others in takeover decisions during autonomous driving
Source: Heliyon. 2024 Apr 16;10(9):e29616. doi: 10.1016/j.heliyon.2024.e29616 (PMC11064069; doi:10.1016/j.heliyon.2024.e29616)
Supplement: Multimedia component 1 [file mmc1.docx]

**Supplementary materials**

**Table S1.** Takeover decisions according to survey type and situation.

| **(A). AV planning to sacrifice the driver** | | | |
| --- | --- | --- | --- |
| Group | No takeover | Takeover | Total |
| Video-based survey | | | |
| Drove off cliff | 31 | 109 | 140 |
| Drove into pedestrians | N/A | 110 | 110 |
| Total | 31 | 219 | 250 |
| Group | No takeover | Takeover | Total |
| Text-based survey | | | |
| Drove off cliff | 28 | 93 | 121 |
| Drove into pedestrians | N/A | 129 | 129 |
| Total | 28 | 222 | 250 |
| **(B). AV planning to sacrifice the pedestrians** | | | |
| Group | No takeover | Takeover | Total |
| Video-based survey | | | |
| Drove off cliff | N/A | 107 | 107 |
| Drove into pedestrians | 95 | 48 | 143 |
| Total | 95 | 155 | 250 |
| Group | No takeover | Takeover | Total |
| Text-based survey | | | |
| Drove off cliff | N/A | 84 | 84 |
| Drove into pedestrians | 108 | 58 | 166 |
| Total | 108 | 142 | 250 |

**Table S2.** Role of sex and driving license possession in takeover decisions.

| (A) AV planning to sacrifice the driver | | | | | |
| --- | --- | --- | --- | --- | --- |
|  | | No takeover | Takeover | | Total |
| Male (video/text) | 8/8 | | | 98/98 | 106/106 |
| Female (video/text) | 23/20 | | | 121/124 | 144/144 |
| Total (video/text) | 31/28 | | | 219/222 | 250/250 |
| Have a license (video/text) | 21/19 | | | 153/161 | 174/180 |
| No license (video/text) | 10/9 | | | 66/61 | 76/70 |
| Total (video/text) | 31/28 | | | 219/222 | 250/250 |
| (B) AV planning to sacrifice the pedestrians | | | | | |
|  | No takeover | | | Takeover | Total |
| Male (video/text) | 41/46 | | | 65/60 | 106/106 |
| Female (video/text) | 54/62 | | | 90/82 | 144/144 |
| Total (video/text) | 95/108 | | | 155/142 | 250/250 |
| Have a license (video/text) | 60/71 | | | 114/109 | 174/180 |
| No license (video/text) | 35/37 | | | 41/33 | 76/70 |
| Total (video/text) | 95/108 | | | 155/142 | 250/250 |

**Table S3.** Role of age and driving experience in takeover decisions.

| (A). AV planning to sacrifice the driver | No takeover | Takeover |
| --- | --- | --- |
| Age (SD) – video | 21.8 (2.74) | 23.3 (3.06) |
| Age (SD) – text | 22.8 (3.28) | 23.0 (3.46) |
| Driving distance – km (SD) – video | 224.19 (653.05) | 794.45 (2509.32) |
| Driving distance – km (SD) – text | 1870.07 (5848.24) | 1806.05 (8338.52) |
| (B). AV planning to sacrifice the pedestrians | No takeover | Takeover |
| Age (SD) – video | 22.8 (2.72) | 23.3 (3.23) |
| Age (SD) – text | 22.9 (3.36) | 23.0 (3.25) |
| Driving distance – km (SD) – video | 563.28 (3951.62) | 822.08 (2609.51) |
| Driving distance – km (SD) – text | 1024.41 (3951.62) | 2413.16 (10141.11) |


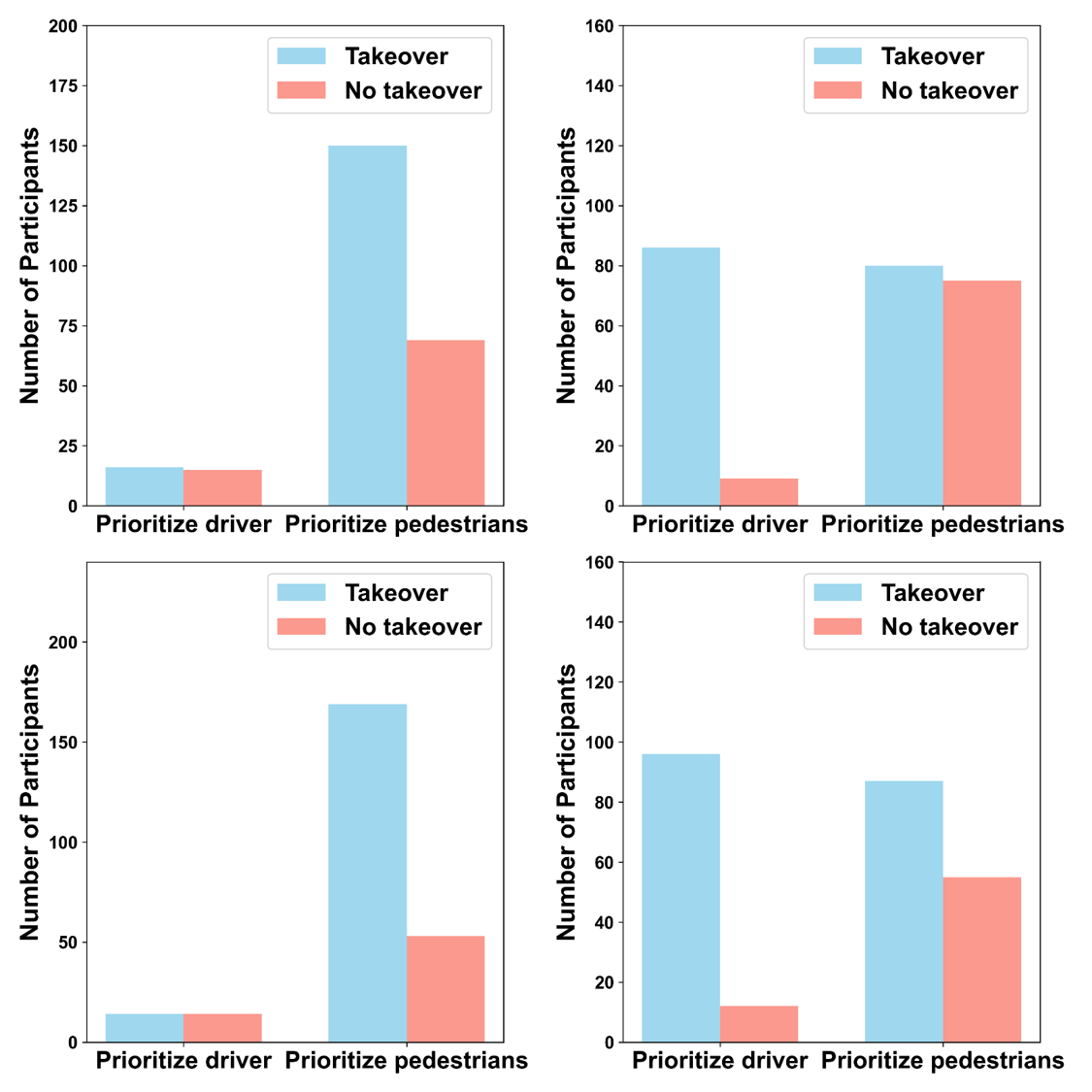


**Fig. S1.** Role of safety priority on the AV in takeover decisions. (A) AV planning to sacrifice driver – video survey (B) AV planning to sacrifice pedestrians – video survey (C) AV planning to sacrifice driver – text survey (D) AV planning to sacrifice pedestrians – text survey


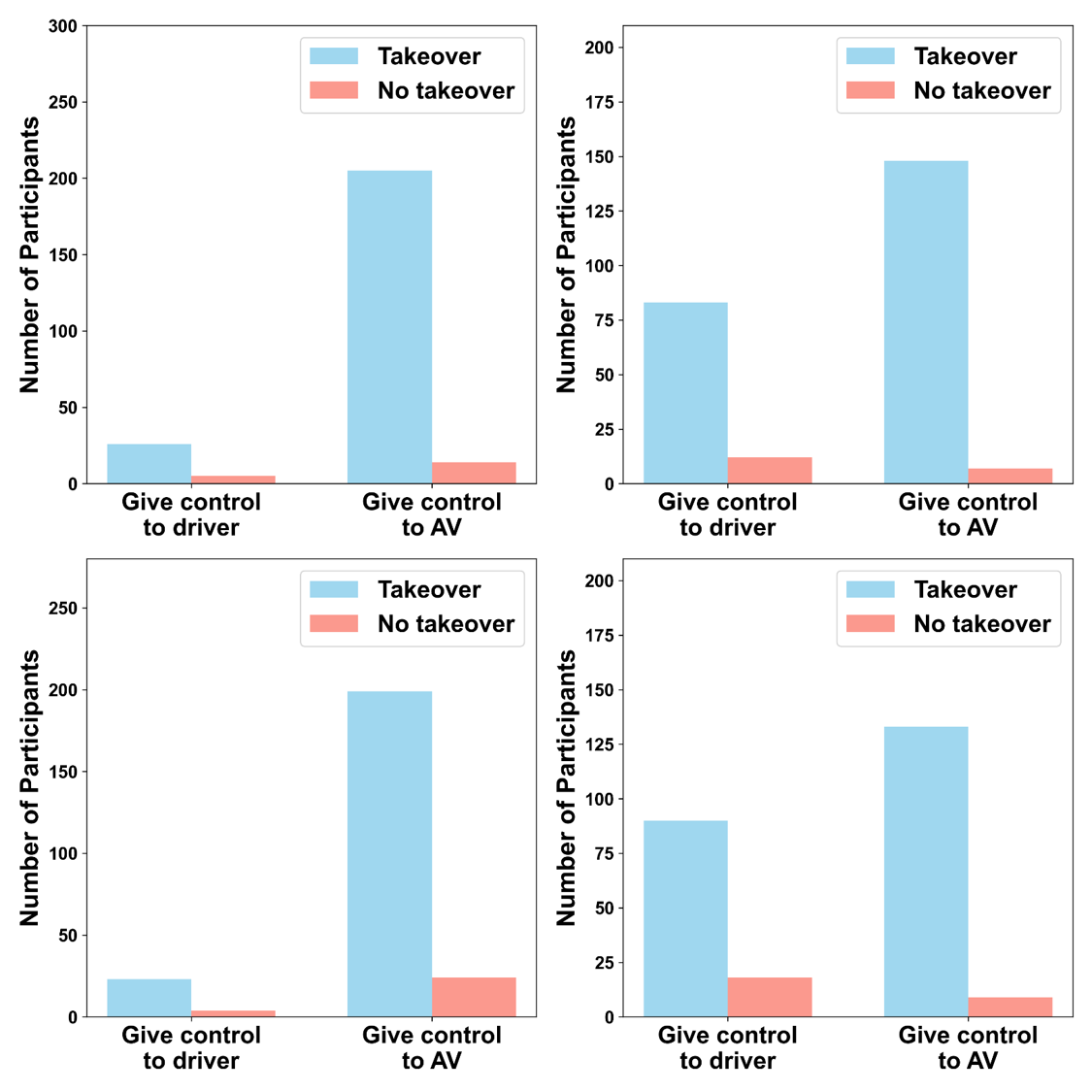


**Fig. S2.** Role of driving control priority on the AV in takeover decisions. (A) AV planning to sacrifice driver – video survey (B) AV planning to sacrifice pedestrians – video survey (C) AV planning to sacrifice driver – text survey (D) AV planning to sacrifice pedestrians – text survey


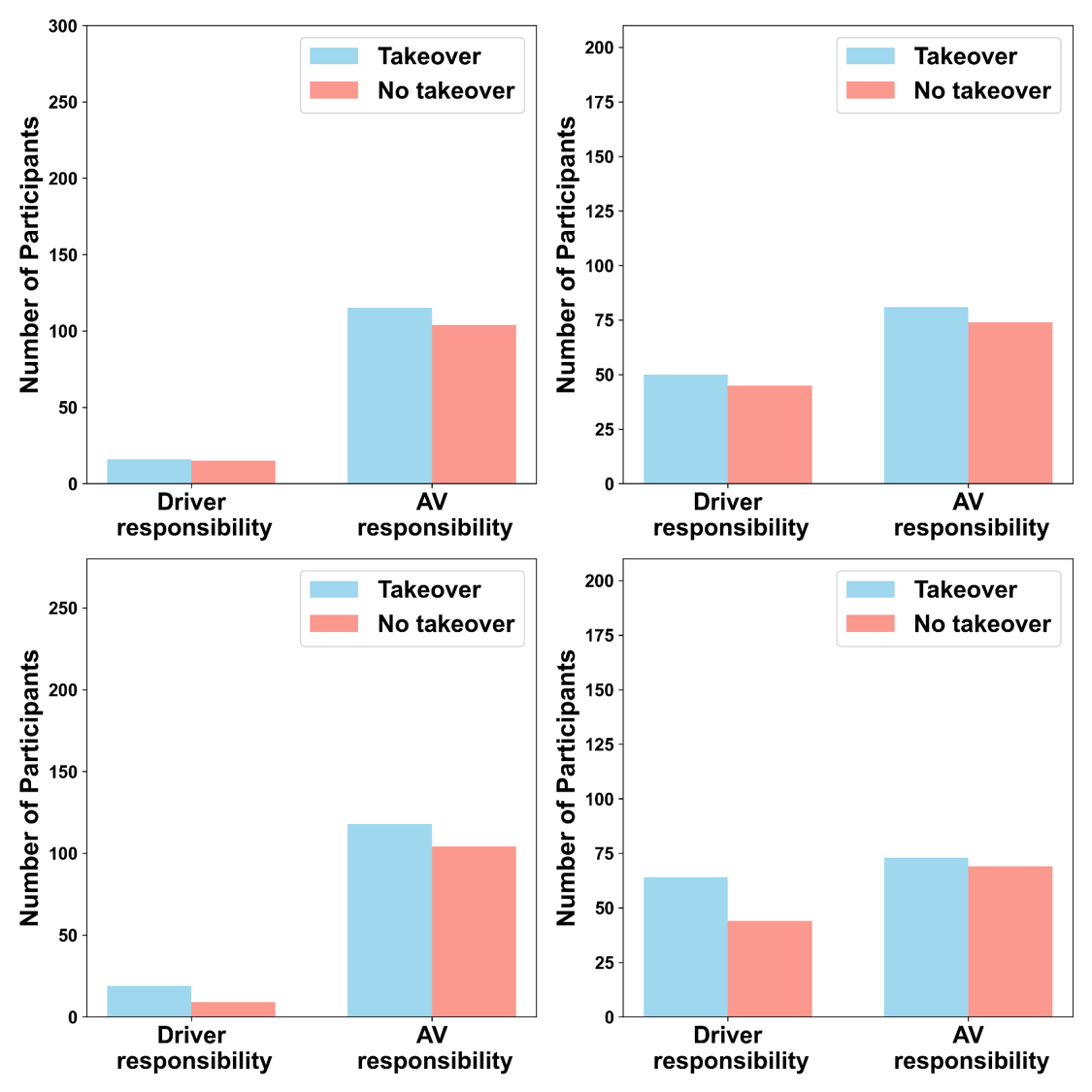


**Fig. S3.** Role of accident responsibility on the AV in takeover decisions. (A) AV planning to sacrifice driver – video survey (B) AV planning to sacrifice pedestrians – video survey (C) AV planning to sacrifice driver – text survey (D) AV planning to sacrifice pedestrians – text survey


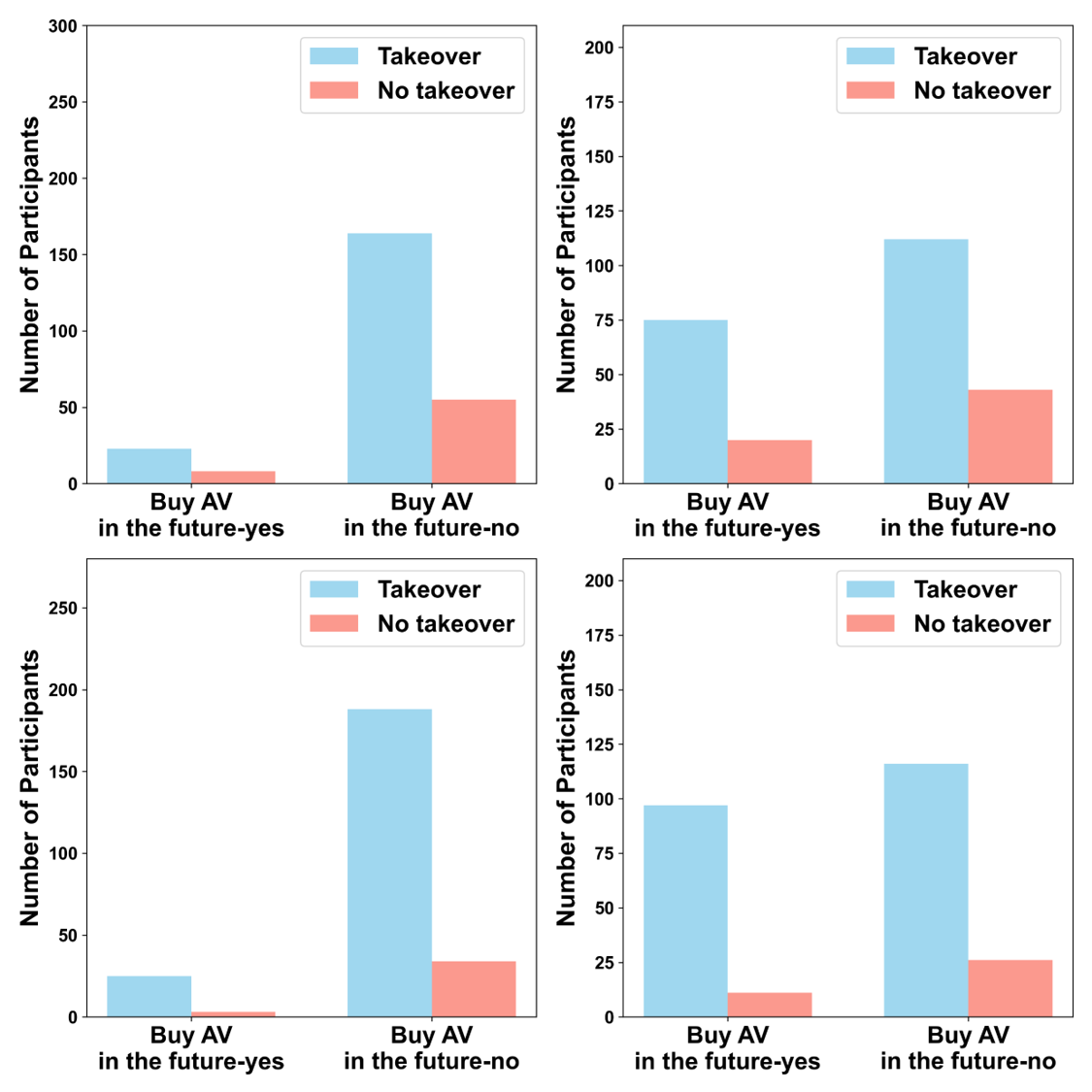


**Fig. S4.** Role of willingness to buy AV in the future on the AV in takeover decisions. (A) AV planning to sacrifice driver – video survey (B) AV planning to sacrifice pedestrians – video survey (C) AV planning to sacrifice driver – text survey (D) AV planning to sacrifice pedestrians – text survey
